# Long-term trajectories of depressive symptoms by military affiliation

**DOI:** 10.1016/j.ssmph.2024.101733

**Published:** 2024-11-29

**Authors:** Elizabeth C. Coppola, Shelley MacDermid Wadsworth, Zoe E. Taylor, Laura Schwab-Reese, Sharon L. Christ

**Affiliations:** a VA Connecticut Healthcare System, West Haven, CT, USA; b Department of Human Development and Family Studies, Purdue University, USA; c Department of Public Health, Purdue University, USA

**Keywords:** Life course, Depressive symptoms, Military service, Trajectories, Latent growth mixture modeling

## Abstract

**Objectives:**

Using a national sample of Americans, this study estimated and compared patterns of depressive symptom trajectories stratified by military service. This study then examined associations between sociodemographic factors theorized to shape entry into military service and trajectory patterns.

**Method:**

Data came from the National Longitudinal Study of Adolescent to Adult Health, a nationally representative study that followed participants from adolescence (1994–1995) through midlife (2016–2018). Latent growth mixture modeling was used to estimate depressive symptom trajectories among civilians (*n* = 17,644) and participants who served in the military (*n* = 1266). Associations between trajectory membership and sociodemographic factors were tested with multinomial regression.

**Results:**

Trajectories were best represented by 4-class linear models. “Low” was the largest class, representing 74.4% of civilians and 70.4% of those who served. The second largest class, “low then increasing,” was comprised of 13.6% of civilians and 19.6% of service members. The third smallest class, “high then decreasing” class, included 8.8% of civilians and 4.5% of service members. An “increasing” class emerged with high depressive symptoms by midlife, comprised of 3.2% of civilians and 5.5% of those who served. Gender and family structure had robust associations with trajectory membership.

**Discussion:**

A larger percentage of those who served were in the “increasing” trajectory characterized by concerningly high depressive symptoms by midlife, underscoring the need for continued screening in depressive symptoms throughout the life course. Associations between family structure and gender on depressive symptoms support calls for conceptualizing family structure as a social determinant of health and continued investment in women's health.

## Highlights

• Best-fitting models had four trajectories, regardless of military affiliation.
• The largest trajectory had low levels of depressive symptoms.
• A trajectory characterized by high depressive symptoms emerged.
• A larger percent of service members were in the trajectory with elevated symptoms.
• Gender and family structure had among the strongest associations with trajectories.

## 1 Introduction

With military service comes many challenges and opportunities that are likely to yield lifelong consequences for mental health. Military life presents stressors that may pose threats to mental health and wellbeing such as role changes, multiple transitions, and the potential for exposure to traumatic events (National Academies of Sciences Engineering and Medicine, 2019). With military service also comes many resources and services that may serve as protective factors by promoting upward social mobility, a sense of belongingness, social support (Spence et al., 2013), and other tangible and intangible benefits. Researchers have identified systematic differences in demographic and family characteristics between civilians and those who serve since the shift to an all-volunteer force in 1973 (Blosnich et al., 2014), raising questions about the characteristics and circumstances that lead to volunteering for military service. These considerations have led the Department of Defense (Department of Defense, 2022) to acknowledge the need to understand how differences in pre-military characteristics— including demographics and family characteristics— shape subsequent health, leading to poorer outcomes for some while others thrive.

Life course perspectives offer a lens for understanding how the transition into military service may alter the life course, leading to qualitatively distinct trajectories for those who enter the military compared to those who enter the civilian workforce or postsecondary education. These perspectives pose that humans develop in socially and psychologically meaningful ways throughout the lifespan with earlier experiences shaping subsequent ones (Elder et al., 2003). Entry into military service has been theorized to shape life course trajectories as a turning point, or subjective change in life direction (Elder, Wang, Spence, Adkins, & Brown, 2010), in which the transition into military service induces discontinuity and may lead to improving or worsening long-term trajectories (Wilmoth and London, 2013). To gain a more complete account of the impact of military service on subsequent health and aging, these propositions also assert the need to examine both the long-term outcomes associated with military service, in addition to the characteristics of individuals and their contexts prior to and after military service (Spiro III et al., 2016). Moreover, “shared” aspects of military life (e.g., combat) have been theorized to impact individuals differently, conditional on their individual and contextual factors (Spiro III et al., 2016).

Individual and contextual factors may also lead individuals to enlist in the military, yielding systematic differences between civilians and those who join the military. The military has been viewed as a bridge to greater opportunity for educational and professional development (Browning et al., 1973) by offering a range of benefits including housing assistance, healthcare, consumer subsidies, childcare, and household maintenance allowances, among others (NASEM, 2019). These benefits may be particularly appealing to individuals whose opportunities are constrained by family disadvantage, who may perceive military service as a pathway to achieve a degree of otherwise unattainable economic independence (Bennett & McDonald, 2013). With military service also comes intangible benefits, such as a greater sense of belonging and camaraderie with the provision of stable, positive interactions and camaraderie with a small group of people (e.g., a military squad), which may motivate enlistment decisions among youth from non-traditional family structures (Spence et al., 2013).

Most individuals successfully cope with the demands of military life (NASEM, 2019); nevertheless, military service during the post-9/11 era have been marked by a growing population of service members and veterans seeking mental health treatment. Service members’ depression diagnoses increased by more than sevenfold during this era (Seal et al., 2009), with more than half of veterans who had recently separated from military service screening positive for probable depression (Aronson et al., 2020). These findings have led to a heightened interest in examining depressive symptom trajectories prior to and throughout military service.

To date, no known efforts have examined heterogeneity in trajectories of depressive symptoms across developmental periods or throughout the transition into military service with consideration of preservice characteristics. To address this gap, we modeled heterogeneous trajectories of depressive symptoms from adolescence through midlife, identifying trajectories of depressive symptoms before and during the transition into—and in some instances, out of—military service. Heterogenous patterns of depressive symptom trajectories were compared by military affiliation, providing information on how the context of military service differentially impacts trajectories of depressive symptoms across life stages. Finally, we examined the role of sociodemographic factors associated with selection into military service and their associations with depressive symptom trajectories as posed by life course theory. We then conclude by discussing how findings inform the DoD's (2022) calls for enhanced resourcing among those who disproportionately bear the burden of past and current hardship based on sociodemographic factors.

## 2 Background

Life course theory poses that human development is a lifelong process in which earlier life experiences, embedded within a sociohistorical context, shape subsequent development (Elder, Wang, Spence, Adkins, & Brown, 2010). This perspective poses that vulnerability is dynamic, which has been supported in research documenting depressive symptoms as following a U-shaped curve (Sinkewicz et al., 2022) in which they are typically elevated during adolescence, decrease throughout the transition into early adulthood, reach their lowest levels during midlife, and then increase again later in life (Mirowsky & Ross, 1992). Changes in depressive symptoms have been theorized to coincide with discrete changes in roles (turning points) and gradual changes associated with the acquisition or relinquishment of roles (transitions) experienced by individuals at different stages of the life course (Elder, Wang, Spence, Adkins, & Brown, 2010; Mirowsky & Ross, 1992). In the context of military service, entry into the military has been characterized as a turning point with the potential to alter long-term patterns of stability and change (trajectories), leading to different outcomes for different individuals (Elder, Wang, Spence, Adkins, & Brown, 2010). Military service has been theorized to yield positive, null, or negative trajectories at different periods within the life course (Spiro III et al., 2016), warranting the need to examine heterogeneity across developmental periods.

Premilitary characteristics such as family structure and family socioeconomic status (SES), have been theorized to shape entry into military service and subsequent, long-term outcomes associated with military service (Spiro III et al., 2016). Adolescents from non-traditional families experience mental health difficulties at disproportionately higher rates than those raised by both biological parents (Melero & Sánchez-Sandoval, 2017) as do adolescents from lower income households (NASEM, 2019). These adolescents may be particularly receptive to military culture and recruitment materials, viewing military service as an opportunity to access a support system, a reliable group of individuals providing them with camaraderie (Spence et al., 2013), a pathway to independent adult life, social mobility, and occupational prestige that is otherwise inaccessible (Kamarck, 2019; Spence et al., 2013). Since military life enables adolescents to exit their family homes and transition into independence at an earlier age than typically allowed in the civilian labor force (Spence et al., 2013), family contextual factors may be less impactful for those who serve in the military.

Additional sources of heterogeneity in the effects of military service on trajectories include sociohistorical context and agency. Military service since the 1973 inception of the all-volunteer force is nonrandom and subject to both self-selection (i.e., individuals exert agency in deciding to enlist) and institutional selection (i.e., screening requirements; Wolf et al., 2013), creating health differentials between civilians and those who serve (Spiro et al., 2016). These selection factors have led to shifts in the demographic composition, resulting in a military that is more racially and ethnically diverse than the general population, yet less diverse with respect to gender and SES (Patten & Parker, 2011; Wolf et al., 2013). These demographic characteristics have also been theorized to contribute to heterogeneity through differential social structuring of life experiences, exposure to stressors, and access to resources (Jones et al., 2019). The DoD has implemented policies, programming, and resources designed to counter these race-, ethnic- and gender-based health disparities observed in a civilian context (NASEM, 2019), although it remains unknown whether associations between these demographics and depressive symptoms differ in a military versus civilian context.

## 3 Heterogeneity in depressive symptom trajectories

Efforts to longitudinally model heterogeneity in depressive symptoms have identified substantial variation in the duration (chronicity), severity, and frequency of reoccurrence (Musliner et al., 2016; Shore et al., 2018), distinguishing those who go on to develop severe depressive symptoms after experiencing low symptoms from those whose symptom levels are chronically high (Eaton, 2011). Most studies of depressive symptom trajectories have used data from general populations (Musliner et al., 2016; Schubert et al., 2017; Sinkewicz et al., 2022; Shore et al., 2018), identifying that whereas most individuals experience low depressive symptoms throughout the life course, others experience chronically elevated symptoms (Lorenzo-Luaces, 2015) or subclinical symptoms with the potential to lead to more severe depression (Cuijpers & Smit, 2004). Only two known studies have examined depressive symptom trajectories in military populations (Coppola et al., 2021; Sampson et al., 2022), suggesting that service members also follow the general population pattern with the largest group displaying low stable depressive symptoms across time. These studies, however, followed service members only through a two- (Coppola et al., 2021) or six-year period (Sampson et al., 2022), despite calls for a dynamic approach to understanding long-term outcomes of military service that warrant longitudinal data spanning across multiple developmental periods (Spiro et al., 2016). Importantly, neither of those studies were able to incorporate prospective data prior to military service, or to compare military and civilian populations, two key innovations of the present study.

## 4 Method

We used data from all five waves of the restricted version of the National Study of Adolescent to Adult Health (Harris, Halpern, Biemer, Liao, & Dean, 2019a), large nationally representative cohort, to model heterogeneous trajectories of depressive symptoms from adolescence (i.e., prior to military service) throughout the transition into military service and into midlife. To be eligible for participation, at baseline (wave I) youth had to be (1) enrolled in 7th through 12th grade during the 1994–1995 academic year and (2) complete an in-school survey in 1994. In wave II, the study only surveyed adolescents who were still enrolled in school. Therefore, all participants completed wave I and most completed wave II prior to entry into the military.

Participants in the prospective cohort study completed a 90-min in-home survey in wave I (1994–1995; 79% response rate; *M*_Age_ = 15.97, *SD* = 1.80) and were then tracked in subsequent, follow-up interviews: in grades 8–12 in wave II (1996; 88.6% response rate; *M*_Age_ = 16.47, *SD* = 1.62), ages 18–26 in wave III (2001–2002; 77.4% response rate; *M*_Age_ = 22.31, *SD* = 1.84), ages 24–32 in wave IV (2008; 80.3% response rate; *M*_Age_ = 28.83, *SD* = 1.82), and ages 33–44 in wave V (2016–2018; 62% response rate; *M*_Age_ = 37.89, *SD* = 1.93).

Add Health originally included 20,745 participants. After eliminating cases that lacked data on military status in at least one wave (*n* = 1835), the final analytical sample contained 17,644 civilians and 1266 participants who served in the military. The average age of enlistment was 19.98 (median: 19; IQR: 3), with over 90% enlisting by age 23. Among the 65.08% of participants who reported either transitioning into veteran status or currently in wave V, 81.04% left military service by age V (*M*_Age_ = 26.07 years old). All procedures were approved by Purdue University IRB (protocol #2019–39).

## 5 Measures

Measures used in the present study are summarized below with more detailed information in supplement S1.

### 5.1 Military status

Participants who have indicated that they served in the military in the past or present in waves III-V were included in the military sample (see supplement S2 for detailed information on the military subpopulation).

### 5.2 Depressive symptoms (waves I-V)

Depressive symptoms were measured using five items from the Center for Epidemiologic Studies Depression Scale (CES-D; Radloff & Sawyer, 1977), in which respondents reported how frequently they experienced specific thoughts or emotions over the past week using a scale of 0–3. These items have been previously identified as valid and reliable measures of depressive symptoms (Perreira et al., 2005) and demonstrated strong reliability in these data (*α* = .96 in wave 1; *α* = .99 in waves II-V) We generated factor score means in which higher values reflected higher levels of depressive symptoms. Factor scores weight responses to individual items according to factor loadings, yielding advantages to summed or averaged scores due to its use of maximum likelihood, ability to retain information from all respondents, and enhanced reliability, especially in the context of missing data (Estabrook & Neale, 2013). Average levels of depressive symptoms at wave I were 0.64 and 0.57 for civilians and service members (see supplement S3 for mean factor scores in subsequent waves).

### 5.3 Sociodemographic variables (wave I)

We measured race and ethnicity as self-identification as non-Hispanic White (reference), Non-Hispanic Black or African American, or Hispanic (herein White, Black, and Hispanic). Using self-report data, we categorized family structure as being raised by two biological parents (reference), in a two-parent household with a stepparent, or in a single-parent household. Parents of Add Health participants reported on their own and their partners' highest level of educational attainment; we supplemented missing data using participants' reports on their parents' education level. Consistent with other approaches in the literature (Jang & Kim, 2023), parental educational attainment was categorized to reflect years of schooling completed. We then conducted a sensitivity analysis using an indicator variable representing parental college attainment (bachelor's degree or higher), as opposed to years of schooling completed (Jang & Kim, 2023). Participants' parents reported their combined household income from all sources in the previous year in $1000 units. Following Elder and colleagues (2010), we log-transformed household income to reduce skewness.

## 6 Analysis

We generated depressive symptom factor scores at each wave by scaling to an item that demonstrated invariance across time and groups. Factor scores yield advantages to summed or averaged scores by relying on full-information maximum likelihood (FIML) to enable the retention of information from all respondents, regardless of item-level missingness, and by weighting responses to individual items according to factor loadings (Estabrook & Neale, 2013).

We used latent growth mixture modeling (LGMM), an extension of conventional growth modeling, to model depressive symptom trajectories from adolescence through midlife separately for those with and without a military service history. LGMM relaxes the assumption of a single population trajectory by allowing individual trajectories to vary around distinct growth curve means and variances and enabling the modeling of longitudinal change in depressive symptoms and latent depressive symptom intercepts and slopes within latent trajectory classes. This approach yields a latent trajectory class variable that represents unobserved subpopulations of growth, enabling the empirical identification of population subgroups with separate growth curves that are distinct from each other.

We determined the optimal number of trajectory classes by progressively increasing the number of trajectories by one class per each shape of change (i.e., linear and quadratic) and evaluating whether the addition of a trajectory class yielded conceptually and statistically superior solutions (Masyn, 2013). Model selection was guided by a holistic evaluation of multiple statistics (Masyn, 2013) including Bayesian information criteria (BIC), sample-size adjusted BIC (SSA-BIC; lower is better), and Akaike information criterion (AI); classification quality was evaluated with entropy (values closer to 1 indicates fewer classification errors; van de Schoot, Sijbrandij, Winter, Depaoli, & Vermunt, 2017). We prioritized substantive meaningfulness and parsimony during model selection (Muthén, 2003). Analytic procedures followed the best practices checklist established by van de School and colleagues (2017) (see checklist in supplement S4; S15 contains technical details on class enumeration). After selecting the best-fitting model, we drew comparisons by subpopulations on the following dimensions: configural (number of distinct trajectory classes), structural (intercepts and slopes across populations), distributional (proportion of observations in each class), and predictive (associations between sociodemographic factors and depressive symptom trajectory class) similarity (Morin et al., 2016).

We extracted an indicator variable representing latent trajectory class based on individuals’ most likely class membership in best-fitting models. Latent class trajectory membership was regressed on sociodemographic factors to examine the associations of sociodemographic factors with trajectory class. Associations were represented by odds ratios (OR), or change in likelihood of membership in one trajectory class versus a comparison trajectory as a function of sociodemographic factors. Following other approaches in the literature (Miller et al., 2023), effects (*Note*. We use effect to match the methodological nomenclature only, not to suggest causal associations) were interpreted when odds ratios represented at least a small effect size (i.e., <0.70 or >1.40; Chen et al., 2010) or when *p* < .05 (note we use effect to match the methodological nomenclature only, not to suggest causal associations).

Models used Mplus 8.8 (Muthén & Muthén, 2014) with full information maximum likelihood estimation (FIML) to allow for the inclusion of participants with missing data. FIML is unbiased when data are missing at random, is less biased than other missing data approaches, can mitigate selective attrition, and reduces nonresponse bias (Enders, 2010). Missing data ranged from 0% to 0.89% on all variables except for father's educational attainment (24.58%), and household income (27.04%). All models corrected for school-level clustering with a sandwich variance estimator and for unequal sample selection using wave 1 sampling weights. Time was indexed by participants' average age centered at wave 1 (15.67 years old). All analyses were conducted separately by military affiliation.

## 7 Results

Table 1 presents summary statistics for key variables and covariates. Relative to civilians, a larger percentage of those who served in the military were male and raised in either a stepparent or single-parent household. Subpopulations did not differ by race, parental educational attainment, household income, or age at baseline.

**Table 1:** Population (weighted) sociodemographic by group.

	Combined Population (*n* = 18,910)	Military (*n* = 1266)	Civilian (*n* = 17,644)	Test Statistic^a^	*p*-value
Population size estimate	22,212,539	1,463,538.2	20,670,998		
% Male	50.92%	82.23%	48.71%	−16.92	<0.001
Race					
% White	57.40%	57.22%	57.42%	1.935	0.380
% Black	22.78%	21.72%	22.85%		
% Hispanic	18.38%	18.91%	18.43%		
Education - Mother	13.18	12.87	13.04	0.34	0.184
Education - Father	13.02	13.12	13.19	0.56	0.573
Family Structure					
% 2 Bio Parents	57.64%	52.16%	58.02%	27.773	<0.001
% Stepparents	10.82%	15.29%	10.50%		
% Single Parent	31.00%	31.34%	30.98%		
Income (Unit = $1000)	45.44	44.98	45.48	0.22	0.827
_*M*_Age (Wave I)	15.67	15.98	15.96	−0.21	0.836

a Used weighted logistic regression for gender, parental educational attainment, income, and age, chi-square for race and ethnicity.

Baseline depressive symptoms were not significantly higher among those who did not participate in waves II (*t* = −1.56, *p* = .12), III (*t* = −0.86, *p* = .39), or IV (*t* = −0.61, *p* = .54), although those with higher baseline depressive symptoms were less likely to participate in wave V (*t* = −4.36, *p* < .001). *T*-tests indicated that civilians had higher average depressive symptoms than did those who had served in the military in waves I-IV (*p* < .001), although depressive symptoms did not significantly differ by military affiliation in wave V (see Supplemental Table S1).

## 8 Configurations of trajectories across populations

**Fig. 1:**
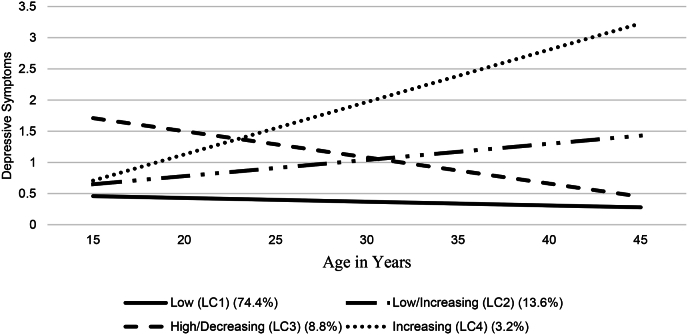
Trajectory classes for the 4-class linear solution among civilians.

**Fig. 2:**
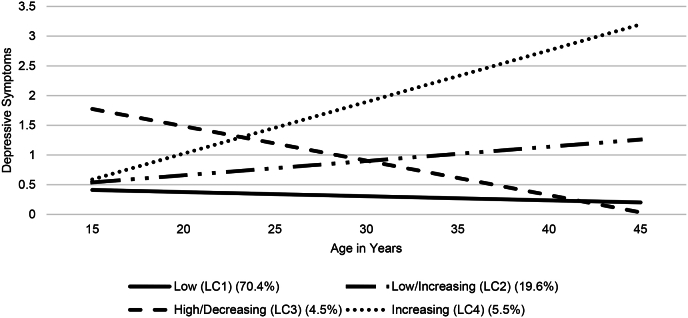
Trajectory Classes for the 4-class Linear Solution Among Those who Served.

Best-fitting models contained four linear trajectories for both subpopulations, indicating configural similarity (see Fig. 1, Fig. 2). Similarities and differences in baseline depressive symptoms (i.e., intercepts) and change in depressive symptoms (i.e., slopes), class proportions, and associations between sociodemographic factors and depressive symptom trajectories by military affiliation are detailed below.

### 8.1 Structural and distributional dissimilarity

Civilians in the “low” (LC1) and “low then increasing” (LC2) trajectories had significantly higher baseline levels of depressive symptoms, relative to corresponding trajectories among those who served. Those who served in the “high then decreasing” (LC3) trajectory had significantly higher baseline depressive symptoms and significantly larger decreases in depressive symptoms across time than did civilians in the “high then decreasing“ (LC3; see Table 2).

**Table 2:** Depression intercepts, slopes, and significant differences across groups.

Intercepts	Military (*n* = 1266)				Civilian (*n* = 17,644)					
	*B*	*p*	CI		*B*	*p*	CI		*B* Difference^a^	*p*
			LL	UL			LL	UL		
Low (LC1)	0.412	<0.001	0.379	0.445	0.459	<0.001	0.443	0.475	−0.0467	<0.001
Low/Increasing (LC2)	0.539	<0.001	0.452	0.625	0.650	<0.001	0.612	0.682	−0.1112	<0.001
High/Decreasing (LC3)	1.774	<0.001	1.498	2.050	1.710	<0.001	1.657	1.763	0.0642	0.037
Increasing (LC4)	0.588	<0.001	0.436	0.740	0.709	<0.001	0.614	0.805	−0.1215	0.136

Slope	*B*	*p*	CI		*B*	*p*	CI		*B* Difference	*p*
			LL	UL			LL	UL		

Low (LC1)	−0.007	<0.001	−0.009	−0.005	−0.006	<0.001	−0.007	−0.005	0.0010	0.259
Low/Increasing (LC2)	0.024	<0.001	0.019	0.029	0.026	<0.001	0.024	0.027	0.0015	0.175
High/Decreasing (LC3)	−0.058	<0.001	−0.074	−0.043	−0.042	<0.001	−0.045	−0.039	0.0065	0.003
Increasing (LC4)	0.087	<0.001	0.075	0.098	0.084	<0.001	0.079	0.088	0.0036	0.223

a Differences reflect civilians' estimates minus estimates from those who served in the military.

The largest trajectory class for both groups, “low“ (LC1), represented 74.4% of civilians (*n* = 13,127) and 70.4% of those who served (*n* = 892). All participants in this class had low levels of depressive symptoms (i.e., below 0.5 on a 4-point scale) throughout the duration of the study, corresponding to an average of experiencing depressive symptoms less than 1–2 days a week, and similar decreases in depressive symptoms throughout the study period.

The next largest class for both groups, “low then increasing” (LC2), represented 13.6% of civilians (*n* = 2397) and 19.6% of those who served (*n* = 248). Participants in the “low then increasing” (LC2) trajectory had low average levels of baseline depressive symptoms (i.e., near 0.5 on a 4-point scale). Depressive symptoms increased at similar rates from adolescence through mid-life across both subpopulations.

Participants in “higher then decreasing” (LC3) trajectory had the highest average levels of baseline depressive symptoms and the steepest decreases in depressive symptoms across development for both subpopulations. Those who served had steeper decreases in depressive symptoms than civilians (1.74 vs. 1.26 point decreases across the study period). Those in the “high then decreasing” (LC3) trajectory represented 4.5% of those who served (*n* = 57) and 8.8% of civilians (*n* = 1554). Those who served in the “low then decreasing” trajectory (LC3) had higher depressive symptoms than those who served in the “low” trajectory (LC1) by age 45. Civilians in the “high then decreasing” trajectory (LC3) had the lowest depressive symptoms by the end of the study period (i.e., age 45), relative to civilians in all other trajectory classes.

Participants in the “increasing” trajectory (LC4) experienced baseline depressive symptoms averaging less than 1–2 days a week (0.547 and 0.644 for those with and without military experience, respectively). Depressive symptoms increased sharply (by 2.61 and 2.52 on a four-point scale among those who served and civilians, respectively) across development, reaching an average of experiencing depressive symptoms 5–7 days a week by age 45. This class represented the smallest proportion of civilians (3.2%; *n* = 566) and third smallest proportion of those who served in the military (5.5%; *n* = 69).

### 8.2 Predictive dissimilarity

Associations between class membership in relation to the “low” trajectory class (LC1) are detailed below and in Table 3. There was limited evidence of predictive similarity across subpopulations.

**Table 3:** Multinomial regression results predicting trajectory class membership relative to low depressive symptoms (LC1).

Military (*n* = 1266)	Low/Increasing (LC2) vs Low (LC1)			High/Decreasing (LC3) vs Low (LC1)			Increasing (LC4) vs Low (LC1)		
	OR	*p*	95% CI	OR	*p*	95% CI	OR	*p*	95% CI
Male	1.083	0.728	[0.685, 1.720]	0.528	0.120	[0.236, 1.181]	0.606	0.160	[0.301, 1.220]
Race (REF = White)									
Black	1.041	0.874	[0.634, 1.708]	0.656	0.347	[0.272, 1.580]	0.487	0.157	[0.180, 1.132]
Hispanic	1.117	0.718	[0.614, 2.029]	0.863	0.805	[0.270, 2.763]	1.289	0.595	[0.506, 3.282]
Income	1.134	0.418	[0.836, 1.538]	1.592	0.183	[0.803, 3.155]	1.292	0.325	[0.776, 2.151]
Mother Education	0.983	0.768	[0.874, 1.104]	0.957	0.600	[0.811, 1.129]	0.982	0.828	[0.841, 1.148]
Father Education	1.023	0.689	[0.915, 1.143]	0.839	0.061	[0.699, 1.008]	1.012	0.913	[0.818, 1.251]
Family Structure (REF = 2 Bio Parents)									
Single Parent	1.345	0.207	[0.849, 2.133]	1.717	0.234	[0.705, 4.183]	1.298	0.535	[0.569, 2.965]
Stepparents	0.810	0.483	[0.449, 1.461]	1.214	0.773	[0.325, 4.533]	1.888	0.163	[0.774, 4.610]

Civilians (*n* = 17,644)	OR	*p*	95% CI	OR	*p*	95% CI	OR	*p*	95% CI

Male	0.696	<0.001	[0.624, 0.775]	0.423	<0.001	[0.362, 0.493]	0.504	<0.001	[0.383, 0.658]
Race (REF = White)									
Black	1.055	0.561	[0.881, 1.262]	1.025	0.735	[0.861, 1.236]	1.017	0.925	[0.721, 1.433]
Hispanic	1.031	0.763	[0.846, 1.256]	1.021	0.818	[0.853, 1.223]	0.969	0.853	[0.698, 1.346]
Income	0.991	0.877	[0.889, 1.106]	0.966	0.585	[0.854, 1.093]	1.063	0.524	[0.880, 1.285]
Mother Education	0.979	0.182	[0.948, 1.010]	1.010	0.653	[0.968, 1.054]	0.979	0.436	[0.928, 1.033]
Father Education	1.024	0.193	[0.988, 1.062]	0.994	0.758	[0.953, 1.035]	0.982	0.610	[0.916, 1.053]
Family Structure (REF = 2 Bio Parents)									
Single Parent	1.183	0.046	[1.003, 1.393]	1.966	<0.001	[1.652, 2.289]	1.431	<0.002	[1.145, 1.790]
Stepparents	0.994	0.953	[0.805, 1.227]	1.789	<0.001	[1.398, 2.266]	1.374	0.072	[0.972, 1.941]

Being female or raised in a single parent or stepparent family was associated with riskier trajectories across all classes for civilians. Male civilians were 31%, 57%, and 49% less likely to be in the “low then increasing” (LC2), “high then decreasing” (LC3), or “increasing” (LC4) trajectory classes, respectively, compared to the “low” depressive symptom class (LC1). Relative to civilians raised by both biological parents, civilians raised by single parents were 18%, 96%, and 43% more likely to be in “low then increasing” (LC2), “high then decreasing” (LC3), or “increasing” (LC4) trajectory classes, respectively, compared to the “low” trajectory class (LC1). Civilians raised by stepparents were 78% more likely than civilians in families with both biological parents to be in the “high then decreasing” (LC3) trajectory than in the “low” trajectory class (LC1).

No effects among those who served were significant at the *p* < .05 level, although several effect sizes met the >1.40 or < 0.70 or criteria (Chen et al., 2010). Males who served were 46.3% less likely to be in “higher than decreasing” (LC3) and 39% less likely to be in the “increasing” (LC4) trajectory than females. Those raised by single parents were 71% more likely to be in the “higher than decreasing” (LC3) trajectory and those raised by stepparents were 88.8% more likely to be in the “increasing” (LC4) trajectory than in the “low” (LC1) trajectory. Relative to white participants, Black participants were 34% and 51% less likely to be in the “high then decreasing” (LC3) or “increasing” (LC4) trajectories than in the “low” trajectory. Participants from higher income households were 59% more likely to be in the “high then decreasing” (LC3) trajectory than in the “low” (LC2) trajectory.

We conducted a sensitivity analysis using an indicator of parental college attainment (instead of years of schooling completed) to determine whether findings were robust to analytic choices regarding the operationalization of a key independent variable. Associations were robust to this alternative operationalization of parental educational attainment (see Supplementary Table S16).

## 9 Discussion

In the present study, which is one of the first to use prospective data to model depressive symptoms among military service members across the life course, we compared trajectories of depressive symptoms by military affiliation. The best-fitting model had the same number of classes and shape of change regardless of military affiliation. However, there were notable differences by military affiliation with respect to depressive trajectory class structures (i.e., intercepts and slopes), distributions (i.e., class proportions), and associations between trajectories and sociodemographic factors. Such differences are suggestive of military service as a turning point.

Perhaps the most notable finding involved the riskiest trajectory class, “increasing” (LC4). A larger proportion of those who served displayed the “increasing” trajectory (LC4) relative to civilians (5.5% vs. 3.2%), providing evidence of distributional dissimilarity. This trajectory represented the smallest proportion of civilians and—in a departure from other findings (Musliner et al., 2016; Shore et al., 2017)–the third largest proportion of those who served in the military. Whereas there is some evidence of similar population averages in depressive symptoms between civilians and those who served (Orak et al., 2021), findings from the present study suggest that concerningly high depressive symptoms were concentrated among a disproportionately large subgroup (LC4) of those who served. By using the full range of depressive symptoms, as opposed a cut score or clinical diagnosis, and by modeling heterogeneity, as opposed to a population average, we were able to acknowledge the full continuum of negative, null, and positive outcomes while enabling the identification of a disproportionately large subgroup characterized by concerningly high depressive symptoms. Such nuances are otherwise missed when examining only population averages.

The riskiest trajectory (LC4 [‘increasing’]) went on to report the highest levels of depressive symptoms by midlife regardless of military affiliation, despite having unremarkable depressive symptoms at baseline. Elevated depressive symptoms during specific developmental periods (i.e., at baseline in “high then decreasing” [LC3] or at midlife in “increasing” [LC4] trajectories) may reflect changes in experiences, roles, and opportunities individuals experience at different stages of the life course. Increases in depressive symptoms in midlife (e.g., “increasing” trajectories [LC4]) may suggest sleeper effects in which the consequences of stress exposure do not manifest until a threshold is met (Wade et al., 2020) or cumulative processes that accentuate trajectory differences in later developmental periods (Elder et al., 2003). Collectively, these findings point to the need for continued monitoring for depressive symptoms throughout the life course, as concerningly high depressive symptoms in the “increasing” trajectories (LC4) manifested during midlife despite low depressive symptoms in earlier developmental periods.

Most participants followed “low” (LC1) trajectories characterized by low depressive symptoms, regardless of military affiliation (74.40% of civilians and 70.42% of those who served). These findings identified that most of those who served did not display elevated depressive symptoms despite exposure to stressors of military life such as frequent relocations, demanding work hours, and the potential for deployment (NASEM, 2019). These findings counter negative stereotypes (i.e., the belief that most of those who serve have mental health challenges) that are ultimately harmful to service members and veterans (NASEM, 2019), underscoring the continued need to challenge inaccurate stereotypes about this population.

With respect to predictive similarity, family structure and gender was associated with trajectory membership across all trajectories for civilians with associations in expected directions (i.e., female civilians and those raised in non-traditional family structures were more likely to be in riskier trajectories). Effects of being male and raised in a non-traditional family structure on trajectory membership were notable among civilians, yet smaller among those who served, suggesting that perhaps sociodemographic factors are less relevant in a military context. Being Black was associated with reduced odds of being in either a “high then decreasing” (LC3) or “increasing” (LC4) trajectory, relative to “low” (LC1), raising the possibility that being Black is associated with less riskier trajectory membership in a military context. Possibly women and racial minority populations may glean more benefits from the meritocratic structure of military life, equal employment opportunity provisions (Kamarck, 2019), universal access to DoD supports and services (NASEM, 2019), and the perceived occupational prestige that come with military service (Lundquist, 2008). Relatively large effect sizes associated with gender suggests that perhaps hardship associated with gender (e.g., gendered harassment, discrimination, or sexual violence) was not fully remediated or prevented. Consistent with other findings in the literature (Bromet et al., 2011; Elovainio et al., 2012), girls and women disproportionately bear the burden of depressive symptoms**,** indicating that gender differences in depressive symptoms represent a key health disparity. These findings set the foundation for a more extensive gender-stratified analysis on depressive symptoms across development.

Family structure yielded the largest effects regardless of military affiliation, building evidence for family structure as an important socially stratified grouping (Russell et al., 2018). Associations between being raised in single-parent families and membership in the “high then decreasing” (LC3) trajectory were among the strongest for those who served and they also larger decreases in depressive symptoms relative to their civilian counterparts, despite higher baseline depressive symptoms. These results suggest that perhaps military service was particularly beneficial for those raised in single-parent families, who may have gleaned a greater sense of belonging from military service. Previous research established that the link between family structure and military enlistment was explained by social isolation in single parent, but not stepparent, families (Spence et al., 2013), which has been attributed to a heightened desire among those raised in single parent families to have positive, stable interactions with a small group of people such as those within a military unit (i.e., the belongingness hypothesis; Baumeister et al., 2000). Add Health unfortunately did not collect information on enlistment decisions or constructs related to the belongingness hypothesis (e.g., camaraderie, social bonds, and esprit de corps; NASEM, 2019); nevertheless, future work should examine how these factors are potentially protective with respect to subsequent depressive symptoms among those who serve.

## Strengths, limitations, and future directions

With the present study come many strengths, including the use of a probability sample to produce nationally representative estimates of trajectories across a quarter of a century. This approach optimizes external validity, increasing confidence in the extent to which these findings can be generalized to the populations of interest. The prospective longitudinal design enables both the study of the development of depressive symptoms prior to and during military service, which is essential to better understanding temporal associations between military service and depressive symptoms (Smith, & Millennium Cohort Study Team, 2011). Moreover, our analytic approach acknowledged the full continuum of depressive symptoms, enabling the study of negative, null, and positive outcomes throughout the lifespan (Spiro et al., 2016). The inclusion of civilians participating in all waves of Add Health enabled comparisons to be drawn, further advancing the understanding of differences by military affiliation. Add Health participants are protected by a Certificate of Confidentiality issued by the Department of Health and Human Services (HHS), which has been demonstrated to improve data quality by mitigating biases associated with overreporting to gain or maintain disability benefits (Smith et al., 1999) and concerns surrounding the disclosure of depressive symptoms.

The present study is not without limitations. Despite high response rates (e.g., 80% of the eligible cases completed wave IV), some differential attrition has been identified in Add Health; researchers have since concluded that wave V respondents adequately represent the population who responded in wave I, rendering the impact of differential attrition negligible (Harris et al., 2019b). Our analyses were limited to time-invariant predictors due to model complexity, although future research should explore the role of time-varying factors that are likely to impact trajectories such as cumulative trauma or receipt of DoD and VA resources throughout the life course. Those who served represented a small proportion of the analytical sample relative to civilians, raising the possibility that limited statistical power hampered the ability to detect significant associations among those who served. Effect sizes are insensitive to this limitation; we therefore interpreted odds ratios that corresponded with at least a small effect size (i.e., <0.70 or >1.40; Chen et al., 2010), although future efforts should replicate these findings with a larger subpopulation of those who serve in the military. We were unable adjust for other pre-service mental health conditions as those data were not collected until much later in development (i.e., after wave II). Future complementary research employing variable-centered approaches, paired with data on factors that may confer a degree of protection such as receipt of VA and DoD resources, may inform the identification of mechanisms related to depressive symptoms.

Limitations aside, the identification of heterogeneous trajectories of depressive symptoms by military affiliation can inform health and social policy. Approaches to addressing health disparities through social determinants of health is a key priority of the Biden-Harris Administration (Executive Order No. 13985 2021), with comprehensive federal efforts currently underway through the DOD's Prevention Plan of Action 2.0 (DoD, 2022) and the HHS (De Lew & Sommers, 2022). These efforts acknowledge the need to measure and identify drivers of disparities in mental health as a critical step in promoting health equity. Whereas the DoD (2022) cited the need to consider the burden of hardship based on gender, race, sexual orientation, religion, and ethnicity, the Biden Administration also includes those who live in rural areas, have disabilities, or are affected by persistent poverty (Executive Order 13,985, 2021). Robust effects of family structure and gender on depressive symptoms support calls for conceptualizing family structure as a social determinant of health (Russell et al., 2018) and underscore the need for continued investment in women's health initiatives. HHS, DoD, Veterans Health Administration, and other government agencies should collect data on family structure and examine how they can better serve non-traditional families. Finally, educational efforts and awareness campaigns should aim to build military cultural competence, dispel negative stereotypes, and promote understanding of military and veteran populations among providers, the general public, and educators.

The current study aligns with federal priorities and goals set by the Biden Administration, HHS (De Lew & Sommers, 2022) and the DoD (2022) on using research to identify, target, and eliminate health disparities. By moving beyond comparisons of population averages, we moved towards a more nuanced understanding of patterns associated with military service by documenting trajectories across the full continuum of depressive symptoms and before, during, and—in some instances—after military service, thus providing a novel, more complete account of depressive symptoms among those who served.

## CRediT authorship contribution statement

**Elizabeth C. Coppola:** Writing – original draft, Visualization, Methodology, Formal analysis, Conceptualization. **Shelley MacDermid Wadsworth:** Writing – review & editing, Conceptualization. **Zoe E. Taylor:** Writing – review & editing, Conceptualization. **Laura Schwab-Reese:** Writing – review & editing. **Sharon L. Christ:** Writing – review & editing, Resources, Formal analysis, Conceptualization.

## Ethics

All procedures were approved by Purdue IRB (protocol #2019–39) and all authors were in compliance with all institutional, state, and federal regulations.

## Author's note

The opinions expressed in this article are those of the authors and do not represent the official policy or position of the U.S. Department of Veterans Affairs or the U.S. government. Dr. Coppola's time is funded by the Advanced Fellowship in Medical Informatics through the VA Office of Academic Affiliations. The funding sources had no involvement in the study design, analyses, article preparation, or decision to submit.

## Declaration of competing interest

The authors declare that they have no known competing financial interests or personal relationships that could have appeared to influence the work reported in this paper.

## Data Availability

The authors do not have permission to share data.

## References

[bib1] Aronson K.R., Perkins D.F., Morgan N.R., Bleser J.A., Vogt D., Copeland L.A., Gilman C.L. (2020). Use of health services among post-9/11 veterans with mental health conditions within 90 days of separation from the military. Psychiatric Services.

[bib2] Bennett P.R., McDonald K.B., Wilmoth J.M., London A.S. (2013). Life course perspectives on military service.

[bib3] Blosnich J.R., Dichter M.E., Cerulli C., Batten S.V., Bossarte R.M. (2014). Disparities on adverse childhood experiences among individuals with a history of military service. JAMA Psychiatry.

[bib4] Bromet E., Andrade L.H., Hwang I., Sampson N.A., Alonso J., De Girolamo G., Kessler R.C. (2011). Cross-national epidemiology of DSM-IV major depressive episode. BMC Medicine.

[bib5] Browning H.L., Lopreato S.C., Poston Jr D.L. (1973). Income and veteran status: Variations among Mexican Americans, blacks and anglos. American Sociological Review.

[bib6] Chen H., Cohen P., Chen S. (2010). How big is a big odds ratio? Interpreting the magnitudes of odds ratios in epidemiological studies. Communications in Statistics - Simulation and Computation.

[bib7] Coppola E.C., Christ S.L., Topp D., Southwell K., Bailey K., MacDermid Wadsworth S. (2021). Trajectories of depression symptoms during the process of deployment in military couples. Military Psychology.

[bib8] Cuijpers P., Smit F. (2004). Subthreshold depression as a risk indicator for major depressive disorder: A systematic review of prospective studies. Acta Psychiatrica Scandinavica.

[bib9] De Lew N., Sommers B.D. (2022). Addressing social determinants of health in federal programs. JAMA Health Forum.

[bib10] Department of Defense (2022).

[bib11] Eaton W.W., Tsuang M.T., Tohen K., Jones P.B. (2011). Textbook of psychiatric epidemiology.

[bib12] Elder G.H., Johnson M.K., Crosnoe R., T Mortimer J., Shanahan M.J. (2003). Handbook of the life course.

[bib16] Elder G.H., Wang L., Spence N.J., Adkins D.E., Brown T.H. (2010). Pathways to the all‐volunteer military. Social Science Quarterly.

[bib17] Enders C. (2010).

[bib18] Estabrook R., Neale M. (2013). A comparison of factor score estimation methods in the presence of missing data: Reliability and an application to nicotine dependence. Multivariate Behavioral Research.

[bib20] Harris, K.M., Halpern, C.T., Biemer, P., Liao, D., & Dean, S.C. (2019a). Add Health Wave V documentation: Sampling and mixed-mode survey design Available from:. http://www.cpc.unc.edu/projects/addhealth/documentation/guides/.

[bib21] Harris K.M., Halpern C.T., Whitsel E.A., Hussey J.M., Killeya-Jones L.A., Tabor J., Dean S.C. (2019). Cohort profile: The national longitudinal study of adolescent to adult health (Add health). International Journal of Epidemiology.

[bib22] Jang H., Kim J. (2023). Peers' parental education and cardiovascular disease risk in adulthood: The mediating role of health-related behaviors. Social Science & Medicine.

[bib24] Jones N.L., Gilman S.E., Cheng T.L., Drury S.S., Hill C.V., Geronimus A.T. (2019). Life course approaches to the causes of health disparities. American Journal of Public Health.

[bib25] Kamarck K.N. (2019). https://sgp.fas.org/crs/natsec/R44321.pdf.

[bib26] Lorenzo-Luaces L. (2015). Heterogeneity in the prognosis of major depression: From the common cold to a highly debilitating and recurrent illness. Epidemiology and Psychiatric Sciences.

[bib27] Lundquist J.H. (2008). Ethnic and gender satisfaction in the military: The effect of a meritocratic institution. American Sociological Review.

[bib28] Masyn K.E., Little T.D. (2013). The Oxford handbook of quantitative methods.

[bib29] Miller C.J., Stolzmann K., Dichter M.E., Adjognon O.L., Brady J.E., Portnoy G.A., Iverson K.M. (2023). Intimate partner violence screening for women in the veterans health administration: Temporal trends from the early years of implementation 2014-2020. Journal of Aggression, Maltreatment & Trauma.

[bib30] Mirowsky J., Ross C.E. (1992). Age and depression. Journal of Health and Social Behavior.

[bib31] Morin A.J., Meyer J.P., Creusier J., Biétry F. (2016). Multiple-group analysis of similarity in latent profile solutions. Organizational Research Methods.

[bib32] Musliner K.L., Munk-Olsen T., Eaton W.W., Zandi P.P. (2016). Heterogeneity in long-term trajectories of depressive symptoms: Patterns, predictors and outcomes. Journal of Affective Disorders.

[bib33] Muthén B. (2003).

[bib34] Muthén L.K., Muthén B.O. (2014).

[bib35] National Academies of Sciences, Engineering, and Medicine (2019).

[bib36] Orak U., Kayaalp A., Walker M.H., Breault K. (2021). Resilience and depression in military service: Evidence from the national longitudinal study of adolescent to adult health (Add health). Military Medicine.

[bib37] Patten E., Parker K. (2011). http://www.pewsocialtrends.org/2011/12/22/women-in-the-u-s-military-growing-share-distinctive-profile/.

[bib38] Perreira K.M., Deeb-Sossa N., Harris K.M., Bollen K. (2005). What are we measuring? An evaluation of the CES-D across race/ethnicity and immigrant generation. Social Forces.

[bib40] Radloff, Sawyer L. (1977). The CES-D scale: A self-report depression scale for research in the general population. Applied Psychological Measurement.

[bib41] Russell L.T., Coleman M., Ganong L. (2018). Conceptualizing family structure in a social determinants of health framework. Journal of Family Theory and Review.

[bib42] Sampson L., Cabral H.J., Rosellini A.J., Gradus J.L., Cohen G.H., Fink D.S., Galea S. (2022). Stressful life events and trajectories of depression symptoms in a US military cohort. Scientific Reports.

[bib43] Schubert K.O., Clark S.R., Van L.K., Collinson J.L., Baune B.T. (2017). Depressive symptom trajectories in late adolescence and early adulthood: A systematic review. Australian and New Zealand Journal of Psychiatry.

[bib44] Seal K.H., Metzler T.J., Gima K.S., Bertenthal D., Maguen S., Marmar C.R. (2009). Trends and risk factors for mental health diagnoses among Iraq and Afghanistan veterans using Department of Veterans Affairs health care, 2002–2008. American Journal of Public Health.

[bib45] Shore L., Toumbourou J.W., Lewis A.J., Kremer P. (2017). Child and adolescent mental health.

[bib46] Sinkewicz M., Rostant O., Zivin K., McCammon R., Clarke P. (2022). A life course view on depression: Social determinants of depressive symptom trajectories over 25 Years of Americans' Changing Lives. SSM-Population Health.

[bib47] Smith D.W., Frueh B.C., Sawchuck C.N., Johnson M.R. (1999). Relationship between symptom over-reporting and pre- and post-combat trauma history in veterans evaluated for PTSD. Depression and Anxiety.

[bib48] Smith T.C., Millennium Cohort Study Team (2011). Linking exposures and health outcomes to a large population-based longitudinal study: The Millennium Cohort Study. Military Medicine.

[bib49] Spence N.J., Henderson K.A., Elder G. (2013). Does adolescent family structure predict military enlistment? A comparison of post–high school activities. Journal of Family Issues.

[bib50] Spiro III A., Settersten R.A., Aldwin C.M. (2016). Long-term outcomes of military service in aging and the life course: A positive re-envisioning. The Gerontologist.

[bib51] van de Schoot R., Sijbrandij M., Winter S.D., Depaoli S., Vermunt J.K. (2017). The GRoLTS-checklist: Guidelines for reporting on latent trajectory studies. Structural Equation Modeling: A Multidisciplinary Journal.

[bib52] Wilmoth J.M., London A.S., Wilmoth J.M., London A.S. (2013). Life course perspectives on military service.

[bib53] Wolf D.A., Wing C., Lopoo L.M., Wilmoth J.M., London A.S. (2013). Life course perspectives on military service.

[bib19] Executive Order No. 13985, 3 C.F.R. 7009 (2021). https://www.federalregister.gov/documents/2021/01/25/2021-01753/advancing-racial-equity-and-support-for-underserved-communities-through-the-federal-government.

