# Supplementary material for: Long-term trajectories of depressive symptoms by military affiliation
Source: SSM Popul Health. 2024 Nov 29;29:101733. doi: 10.1016/j.ssmph.2024.101733 (PMC11667064; doi:10.1016/j.ssmph.2024.101733)
Supplement: Multimedia component 1 [file mmc1.docx]

| S1. | | | | |
| --- | --- | --- | --- | --- |
| Variable | Description | Wave | Response Options | Scoring/Categorization |
| Depressive Symptoms | 5 items: How often was the following true during the past week? -You felt that you could not shake off the blues, even with help from your family and your friends. -You felt depressed. -You felt sad. -You were happy. (reverse-coded) -You enjoyed life. (reverse-coded) | Waves I-IV | 0 - Rarely or none of the time [less than 1 day) | The Add Health team did not administer all items in the Center for Epidemiologic Studies Depression Scale (CES-D) across time, and noninvariance (Perreira et al., 2005) and multidimensionality have been reported in the CES-D (Wood, Taylor, & Joseph, 2010); therefore, we derived a unidimensional measure of depressive symptoms and generated factor score means in which higher values reflected higher levels of depressive symptoms. |
|  |  |  | 1- Some or a little of the time (1-2 days) |  |
|  |  |  | 2 - Occasionally or a moderate amount of time (3-4 days) |  |
|  |  |  | 3 - Most or all of the time (5-7 days) |  |
| Military Status | "Are you currently serving in the military?" | Waves III-V | No (0), yes (1) | Created an indicator variable reflecting endorsement of military service in at least one time point: 1 (yes), 0 (no). |
|  | "Have you ever been in the military?" | Waves III-V | No (0), yes (1) |  |
| Gender | 1 item; "Interviewer, please confirm that R's sex is (male) female. (Ask if necessary.)" | Wave I | 1 - Male | Recoded into an indicator value: male (0), female (1). |
|  |  |  | 2 - Female |  |
| Parental Educational Attainment | 2 items; focal parent of Add Health participant responded to two survey items: "How far did you go in school?" and "How far did your current (spouse/partner) go in school?"  Missing data was supplemented with two items administered to Add Health participants: "How far in school did he [resident father] go?" and "How far in school did she [resident mother] go?" | Parent survey administered in wave I; missing data supplemented with adolescent reports in wave I | 1 - 8th grade or less | Following other approaches in the literature (Jang & Kim, 2023), educational attainment was recoded to reflect years of schooling; number of years are shown in parenthesis: eighth grade or less (8); more than eighth grade, but did not graduate from high school (10); high school graduate (12); completed a GED (11.5); went to a business, trade, or vocational school after high school (13); went to college but did not graduate (14); graduated from college or a university (16); professional training beyond a four-year college (18); and never went to school (0).  Mother's educational attainment and father's educational attainment were treated as two separate variables.  Following other approaches in the literature (Jang & Kim, 2023), we then conducted a sensitivity analysis in which parental educational attainment was recoded for each parent to reflect whether they earned a college degree (1 = yes, 0 = no). |
|  |  |  | 2 - More than 8th grade, no high school diploma |  |
|  |  |  | 3 - Went to a business or trade school instead of high school |  |
|  |  |  | 4 - High school graduate |  |
|  |  |  | 5 - Completed a GED |  |
|  |  |  | 6 - Went to business, trade, or vocational school after high school |  |
|  |  |  | 7- Went to college, no degree |  |
|  |  |  | 8 - College degree |  |
|  |  |  | 9 - Post graduate degree |  |
|  |  |  | 10 - Never went to school |  |
| Family Structure | Household roster was established by administering the following item for up to 20 household members: [If REL="father" or "mother":] Which description best fits {NAME}'s relationship to you? | Wave I | Biological father | Recoded to reflect whether participants were raised by two biological parents (REF), in a two-parent household with at least one stepparent or adoptive parent, or a single parent household |
|  |  |  | Step/adoptive mother |  |
|  |  |  | Foster mother |  |
|  |  |  | Other |  |
|  |  |  | Stepparent |  |
|  |  |  | Adoptive father |  |
|  |  |  | Step/adoptive father |  |
|  |  |  | Foster father |  |
|  |  |  | Other |  |
|  |  |  | Biological mother |  |
|  |  |  | Step mother |  |
|  |  |  | Adoptive mother |  |
| Race and Ethnicity | Participants endorsed their race and ethnicity | Wave I | Ethnicity: Are you of Hispanic or Latino origin? | Following the program code repository shared by the Add Health team (Add Health, n.d.), participants who endorsed “Hispanic” were eliminated from any race category and participants who marked “black or African American” were designated as Black and eliminated from other categories.  Responses were recoded into indicator variables: White non-Hispanic (REF), Black non-Hispanic, Hispanic |
|  |  |  | What is your race? White |  |
|  |  |  | What is your race? Black or African American |  |
| Household income | Participants' parents self-reported their combined household income from all resources in the previous year in $1,000 units. "About how much total income, before taxes did your family receive in 1994? Include your own income, the income of everyone else in your household, and income from welfare benefits, dividends, and all other sources. | Parent survey administered in wave I | Free response | Following Elder and colleagues (2010), we log-transformed household income to reduce skewness. |

S2.

Participants were asked “What is the highest military rank you have achieved?” in wave IV. Nearly half of participants indicated their rank as E1-E4; note that warrant officer was combined with officer due as to not risk deductive disclosure related to small cell sizes per terms of the Add Health contract. Data on rank was not collected in wave V.

Participants were asked “In which branch are you currently serving” and “in which branches of the Armed Forces did (have) you serve (served)?” in waves III and IV. The largest proportion of participants indicated that they either formerly or currently serve in the Army.

Among the 67.5% of service members and veterans who provided valid data on experiences in combat in wave IV, 30% indicated that they experienced at least one of four combat-related traumatic experiences: “During your combat deployment, did you ever kill or think you killed someone?” “During your combat deployment, how many times did you engage the enemy in a firefight?” “During your combat deployment, were you wounded or injured “During your deployment, did you see anyone wounded, killed, or dead?” Data on combat experience was not collected in wave V.

| Variable | Raw *n* | Unweighted % | Weighted % |
| --- | --- | --- | --- |
| Rank |  |  |  |
| E1-E4 | 568 | 44.9% | 45.6% |
| E5-E8 | 398 | 31.4% | 31.0% |
| Officer | 69 | 5.5% | 5.3% |
| Unknown | 231 | 18.3% | 18.1% |
| Branch |  |  |  |
| Army | 543 | 42.9% | 40.2% |
| Air Force | 191 | 15.1% | 13.8% |
| Marines | 193 | 15.2% | 15.6% |
| Navy | 275 | 21.7% | 18.4% |
| Coast Guard | 21 | 1.7% | 2.0% |
| Unknown | 43 | 3.4% | 10.0% |
| Combat-related Trauma |  |  |  |
| Combat | 256 | 20.23 | 21.1% |
| No Combat | 599 | 47.31 | 47.0% |
| Unknown | 411 | 32.46 | 32.0% |

S3**.** Average Estimated Depressive Symptom Factor Score Means and Confidence Intervals

| Age | Civilian | | |  | Military | | |
| --- | --- | --- | --- | --- | --- | --- | --- |
|  | Mean | 95% CI | |  | Mean | 95% CI | |
|  |  | LL | UL |  |  | LL | UL |
| 15 | 0.643 | 0.621 | 0.664 |  | 0.575 | 0.528 | 0.621 |
| 20 | 0.576 | 0.560 | 0.591 |  | 0.481 | 0.447 | 0.516 |
| 25 | 0.537 | 0.521 | 0.554 |  | 0.440 | 0.401 | 0.479 |
| 30 | 0.528 | 0.510 | 0.545 |  | 0.451 | 0.407 | 0.495 |
| 35 | 0.546 | 0.527 | 0.566 |  | 0.513 | 0.464 | 0.563 |
| 40 | 0.593 | 0.569 | 0.618 |  | 0.628 | 0.564 | 0.691 |
| 45 | 0.669 | 0.632 | 0.706 |  | 0.794 | 0.700 | 0.888 |

S4

Checklist for best practices for reporting on latent trajectory studies

| ***Criteria*** | ***Location*** |
| --- | --- |
| 1. Is the metric of time used in the statistical model reported?   Yes. *Time was indexed by participants’ age, which was centered at the average age of participants in wave 1 (15.67 years old).* | *Analysis* |
| 2. Is information presented about the mean and variance of time within a wave? *Participants in the prospective cohort study completed a 90-minute in-home survey in wave I (1994-1995; 79% response rate; M_Age_ = 15.97, SD = 1.80) and were then tracked in subsequent, follow-up interviews: in grades 8-12 in wave II (1996; 88.6% response rate; M_Age_ = 16.47, SD = 1.62), ages 18-26 in wave III (2001-2002; 77.4% response rate; M_Age_ = 22.31, SD = 1.84), ages 24-32 in wave IV (2008; 80.3% response rate; M_Age_ = 28.83, SD = 1.82), and ages 33-44 in wave V (2016; 62% response rate; M_Age_ = 37.89, SD = 1.93).* | *Method: Data* |
| 3a. Is the missing data mechanism reported?  *Yes. “We used Mplus 7.1 (Muthén and Muthén 2014) with full information maximum likelihood estimation (FIML) to allow for the inclusion of respondents with data assumed to be missing at random.”* | *Analysis* |
| 3b. Is a description provided of what variables are related to attrition/missing data?  *Baseline depression was not significantly higher among those who did not participate in waves 2 (t = -1.56, p = .12), 3 (t = -0.86, p = .39), or 4 (t = -0.61, p = .54), although those with higher depression symptoms did not participate in wave 5 (t = -4.36, p <.001).* | *Results* |
| 3c. Is a description provided of how missing data in the analyses were dealt with?  *Yes. “We used Mplus 7.1 (Muthén and Muthén 2014) with full information maximum likelihood estimation (FIML) to allow for the inclusion of respondents with data assumed to be missing at random.”* | *Analysis* |
| 4. Is information about the distribution of the observed variables included?  *Yes. See Tables 1 & S-3.* | *Tables 1 & S-3* |
| 5. Is the software mentioned?  *Yes. “We used Mplus 7.1 (Muthén and Muthén 201* | *Analysis* |
| 6a. Are alternative specifications of within-class heterogeneity considered (e.g., LCGA vs. LGMM) and clearly documented? If not, was sufficient justification provided as to eliminate certain specifications from consideration?  Yes. *“We followed trajectory enumeration procedures described in Masyn (2013), which began with a simple structure in which the within-trajectory variance of the intercepts and slopes were fixed to zero and then tested alternative model structures to allow for variance of depression factor scores to vary across trajectories.”* | *Analysis* |
| 6b. Are alternative specifications of the between-class differences in variance-covariance matrix structure considered and clearly documented? If not, was sufficient justification provided as to eliminate certain specifications from consideration?  Yes. *“We considered alternative model specifications that freely estimated depression factor score variances across profiles, but this led to improper or under-identified solutions, suggesting that these models suffered from overparameterization and needed simplification to increase parsimony (Bauer & Curran, 2003; Chen, Bollen, Paxton, Curran, & Kirby, 2001). Thus, we selected the class-invariant, diagonal variance–covariance matrix.”* | *Analysis* |
| 7. Are alternative shape/functional forms of the trajectories described?  Yes.  *We progressively increased the number of trajectories by one for each shape of change (i.e., linear and quadratic) and evaluated whether the addition of a trajectory led to conceptually and statistically superior solutions.* | *Analysis* |
| 8. If covariates have been used, can analyses still be replicated?  Yes. *We derived a latent class indicator variable based on individuals’ most likely class membership from the latent class posterior distribution from the best-fitting unconditional model. Next, we added sociodemographic covariates to latent growth mixture models using latent class indicator variables and misclassification error rates. This approach enables the estimation of sociodemographic covariate effects on trajectory class membership in multinomial logistic regression models after accounting for class uncertainty (Vermunt, 2010; Wickrama, Lee, O’Neal, & Lorenz, 2021)* | *Analysis* |
| 9. Is information reported about the number of random start values and final iterations included?  Yes.  *We started with 100 random starts to and 20 iterations and then doubled the number of random starts and iterations to establish global maxima and avoid convergence on local solutions (McLachlan & Peel, 2000).* | *Analysis* |
| 10. Are the model comparison (and selection) tools described from a statistical perspective?  *Yes*  *“Model selection was guided by a holistic evaluation of multiple statistics (Masyn, 2013) including sample size adjusted Bayesian information criteria (SSA-BIC; lower is better), entropy (reflects classification accuracy; values closer to 1 are better), and the Akaike information criterion (AIC). Incongruence among fit statistics is common (Masyn, 2013), and current methodological guidance is limited and often conflicting regarding the performance of ft indices for mixture models in a variety of study conditions (e.g., Nylund, Asparouhov, & Muthén, 2007; Peugh & Fan, 2013; Tein, Coxe, & Cham, 2013; Tofighi & Enders, 2008), substantive meaningfulness and parsimony was prioritized during model selection, in part through visual inspection of trajectories (Muthén, 2003).”* | *Analysis* |
| 11. Are the total number of fitted models reported, including a one-class solution?  *Yes. We focused on latent growth mixture models with class-invariant, nondiagonal variance–covariance matrix due to improved fit. Five-trajectory linear models had lower AIC, BIC, and SSA-BIC, however the fifth trajectory contained a small group (1% of each subpopulation) that was qualitatively comparable to another trajectory .* | *Results* |
| 12. Are the number of cases per class reported for each model (absolute sample size, or proportion)?  Yes. See Table S5-S7 | *See Table S5-S7* |
| 13. If classification of cases in a trajectory is the goal, is entropy reported?  Yes. See Table S5-S7 | *See Table S5-S7* |
| 14a. Is a plot included with the estimated mean trajectories of the final solution?  Yes. See Figure 1 and Figure 2. | *Figure 1*  *Figure 2* |
| 14b. Are plots included with the estimated mean trajectories for each model?  Yes. See S6-S11 | *S6-S11* |
| 14c. Is a plot included of the combination of estimated means of the final model and the observed individual trajectories split out for each latent class?  Yes. See S15 | *S14* |
| 15. Are characteristics of the final class solution numerically described (i.e., means, SD/SE, n, CI, etc.)?  *Yes. See Table 2* | *Table 2.* |
| 16. Are the syntax files available (either in the appendix, supplementary materials, or from the authors)?  *Authors will provide syntax files upon request.* |  |

S5

Model fit indices of contender models, class-invariant, nondiagonal variance-covariance matrix

| Group | Loglikelihood | AIC | BIC | SSA-BIC | Entropy | Class Proportions |
| --- | --- | --- | --- | --- | --- | --- |
| SMV |  |  |  |  |  |  |
| 1 Class linear | -4394.818 | 8809.635 | 8861.071 | 8829.307 | 1.00 | - |
| 1 Class quadratic | -6376.092 | 12774.185 | 12830.764 | 12795.823 | 1.00 | - |
| 2 Class linear | -4147.511 | 8323.022 | 8395.032 | 8350.562 | 0.86 | 11/89 |
| 2 Class quadratic | -6179.701 | 12389.401 | 12466.555 | 12418.908 | 0.87 | 88/12 |
| 3 Class linear | -4015.301 | 8062.602 | 8144.900 | 8094.076 | 0.93 | 7/7/86 |
| 3 Class quadratic | -6066.485 | 12170.970 | 2268.699 | 12208.346 | 0.91 | 4/11/85 |
| **4 Class linear** | **-3951.762** | **7941.523** | **8039.252** | **7978.899** | **0.89** | **5/5/20/70** |
| 4 Class quadratic | - | - | - | - | - | - |
| 5 Class linear | -3879.663 | 7803.325 | 7916.485 | 7846.602 | 0.92 | 1/4/5/21/69 |
| 5 Class quadratic | - | - | - | - | - | - |
| Civilians |  |  |  |  |  |  |
| 1 Class linear | -63162.492 | 126344.983 | 126422.765 | 126390.986 | 1.00 | - |
| 1 Class quadratic | -6376.092 | 12774.185 | 12830.764 | 12795.823 | 1.00 | - |
| 2 Class linear | -60358.115 | 120742.230 | 120843.346 | 120802.032 | 0.97 | 5/95 |
| 2 Class quadratic | -91733.265 | 183496.529 | 183613.202 | 183565.532 | 0.88 | 11/89 |
| 3 Class linear | -58649.118 | 117330.237 | 117454.687 | 117403.840 | 0.91 | 4/11/85 |
| 3 Class quadratic | - | - | - | - | - | - |
| **4 Class linear** | **-57764.589** | **115567.178** | **115714.962** | **115654.582** | **0.88** | **3/9/14/74** |
| 4 Class quadratic | - | - | - | - | - | - |
| 5 Class linear | -57232.867 | 114509.734 | 114680.853 | 114610.938 | 0.91 | 1/3/8/15/73 |

*Note.* SSA-BIC = Sample Size Adjusted Bayesian Information Criteria.

S6

Model fit indices of contender models, class-invariant, diagonal variance-covariance matrix

| Group | Loglikelihood | AIC | BIC | SSA-BIC | Entropy | Class Proportions |
| --- | --- | --- | --- | --- | --- | --- |
| SMV |  |  |  |  |  |  |
| 1 Class linear | -4471.527 | 8961.053 | 9007.346 | 8978.758 | 1.00 | - |
| 1 Class quadratic | -6450.233 | 12920.467 | 12971.903 | 12940.138 | 1.00 | - |
| 2 Class linear | -4225.742 | 8475.484 | 8537.207 | 8499.089 | 0.91 | 9/91 |
| 2 Class quadratic | -6179.773 | 12387.545 | 12459.556 | 12415.085 | 0.88 | 12/88 |
| 3 Class linear | -4154.887 | 8339.773 | 8416.928 | 8369.281 | 0.84 | 4/23/72 |
| 3 Class quadratic | -6114.177 | 12264.355 | 12356.94 | 12299.763 | 0.86 | 5/18/75 |
| 4 Class linear | -3964.396 | 7964.791 | 8057.376 | 8000.200 | 0.87 | 4/7/16/73 |
| 4 Class quadratic | -6071.161 | 12186.322 | 12299.481 | 12229.599 | 0.89 | 1/6/22/71 |
| 5 Class linear | -3929.044 | 7900.089 | 8008.104 | 7941.399 | 0.90 | 1/4/6/19/70 |
| 5 Class quadratic | -6027.489 | 12106.977 | 12240.711 | 12158.123 | 0.91 | 1/4/9/20/66 |
| Civilians |  |  |  |  |  |  |
| 1 Class linear | -64224.792 | 128467.585 | 128537.588 | 128508.987 | 1.00 | - |
| 1 Class quadratic | -94798.489 | 189616.977 | 189694.759 | 189662.980 | 1.00 | - |
| 2 Class linear | -61098.399 | 122220.798 | 122314.136 | 122276.001 | 0.88 | 13/87 |
| 2 Class quadratic | -91735.212 | 183498.424 | 183607.318 | 183562.827 | 0.88 | 11/89 |
| 3 Class linear | -58758.179 | 117546.358 | 117663.030 | 117615.361 | 0.91 | 4/12/84 |
| 3 Class quadratic | -90921.028 | 181878.056 | 182018.062 | 181960.859 | 0.83 | 5/20/74 |
| 4 Class linear | -58313.054 | 116662.108 | 116802.115 | 116744.912 | 0.86 | 4/4/17/75 |
| 4 Class quadratic | - | - | - | - | - | - |
| 5 Class linear | -89211.251 | 183498.424 | 183607.318 | 183562.827 | 0.87 | 2/3/5/20/70 |

*Note.* SSA-BIC = Sample Size Adjusted Bayesian Information Criteria.

| Group | Loglikelihood | AIC | BIC | SSA-BIC | Entropy | Class Proportions |
| --- | --- | --- | --- | --- | --- | --- |
| SMV |  |  |  |  |  |  |
| 1 Class linear | -5022.325 | 10058.649 | 10094.654 | 10072.419 | 1.00 | - |
| 1 Class quadratic | -6860.676 | 13737.352 | 13778.500 | 13753.089 | 1.00 | - |
| 2 Class linear | -4429.378 | 8878.757 | 8930.193 | 8898.428 | 0.88 | 16/84 |
| 2 Class quadratic | -6310.228 | 12644.457 | 12706.180 | 12668.062 | 0.90 | 14/86 |
| 3 Class linear | -4145.667 | 8317.334 | 8384.201 | 8342.907 | 0.92 | 80/8/12 |
| 3 Class quadratic | -6310.228 | 12652.457 | 12734.755 | 12683.931 | 0.94 | 0/86/14 |
| 4 Class linear | -3992.979 | 8017.958 | 8100.256 | 8049.433 | 0.88 | 68/21/7/4 |
| 5 Class quadratic | -3939.868 | 7917.737 | 8015.465 | 7955.112 | 0.89 | 67/21/6/4/2 |
| Civilians |  |  |  |  |  |  |
| 1 Class linear | -74466.925 | 148947.850 | 149002.297 | 148980.051 | 1.00 | - |
| 1 Class quadratic | -102269.904 | 204555.808 | 204618.033 | 204592.609 | 1.00 | - |
| 2 Class linear | -63759.486 | 127538.972 | 127616.754 | 127584.975 | 0.89 | 20/80 |
| 2 Class quadratic | -93769.711 | 187563.422 | 187656.760 | 187618.624 | 0.89 | 18/82 |
| 3 Class linear | -61245.466 | 122516.932 | 122618.048 | 122576.735 | 0.92 | 5/17/1978 |
| 3 Class quadratic | -93769.711 | 187571.422 | 187695.872 | 187645.025 | 0.93 | 0/18/82 |
| 4 Class linear | -59136.498 | 118304.997 | 118429.447 | 118378.600 | 0.87 | 4/7/23/66 |
| 5 Class quadratic | -59136.498 | 118306.997 | 118439.225 | 118385.200 | 0.89 | 66/23/6/4/0 |
| *Note.* SSA-BIC = Sample Size Adjusted Bayesian Information Criteria. | | | | | | |

S7

Model fit indices of contender models, latent class growth analysis


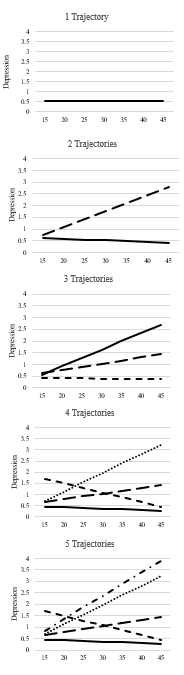

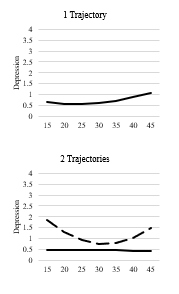


S8

Contender models

Linear (left) and quadratic (right) LGMM class solutions for those without a military service history using a nondiagonal invariant variance-covariance matrix.

S9

Contender models


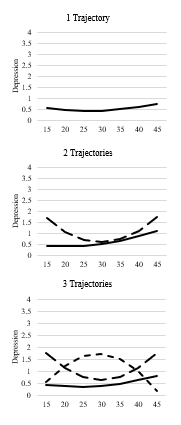

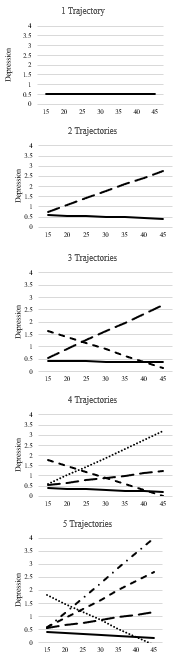


Linear (left) and quadratic (right) LGMM class solutions for those with a military service history using a nondiagonal invariant variance-covariance matrix.


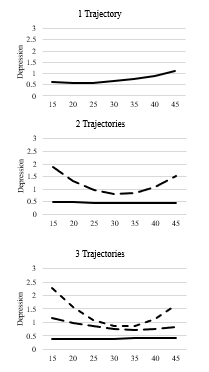

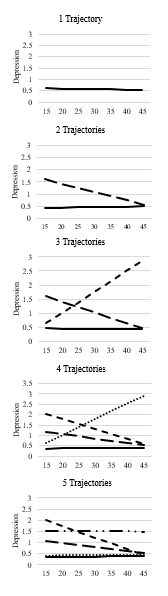


S10

Contender models

Linear (left) and quadratic (right) LGMM class solutions for those without a military service history using a nondiagonal invariant variance-covariance matrix.


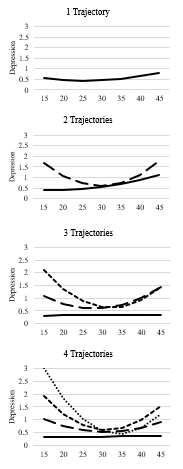

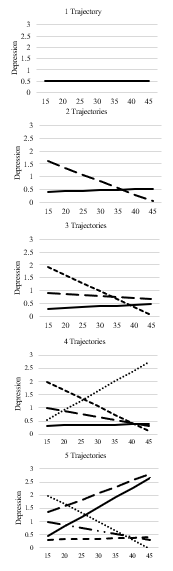


S11

Contender models

Linear (left) and quadratic (right) LGMM class solutions for those with a military service history using a nondiagonal invariant variance-covariance matrix.


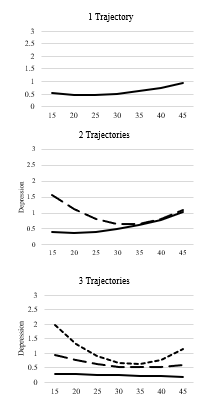

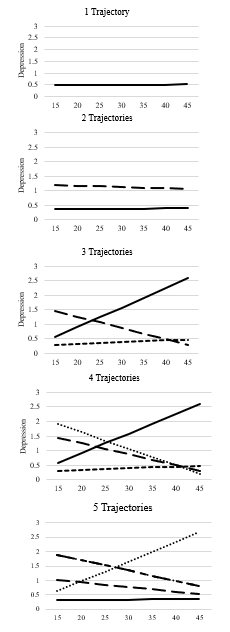


S12

Contender models

Linear (left) and quadratic (right) LCGA class solutions for those with a military service history


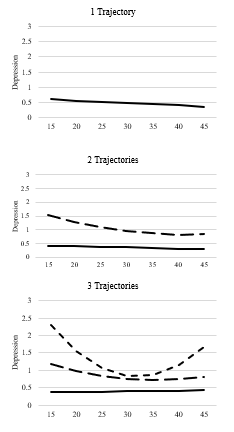

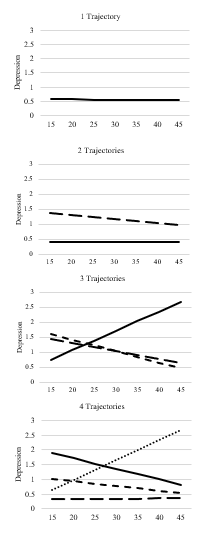


S13

Contender models

Linear (left) and quadratic (right) LCGA class solutions for those without a military service history.


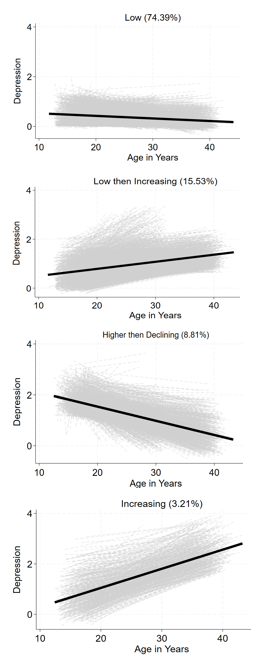

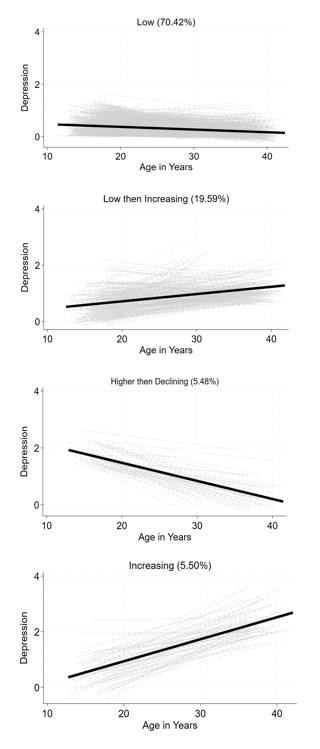


Estimated final class solution means and individual trajectories by class for those with (left) and without (right) a military service history.

S14

Individual trajectories and means by class

S15

We determined the optimal number of trajectory classes by progressively increasing the number of trajectories by one class per each shape of change (i.e., linear and quadratic) and evaluating whether the addition of a trajectory class yielded conceptually and statistically superior solutions. (Masyn, 2013). We started with 100 random starts and 50 iterations and then doubled the number of random starts and iterations to establish global maxima and avoid convergence on local solutions (McLachlan & Peel, 2000). We initially fixed the within-trajectory variance of intercepts and slopes to zero and then tested alternative model structures to allow for variance and covariance of depressive symptom factor scores to vary across trajectories. We considered alternative model specifications that freely estimated mean slopes, intercepts, and covariances across depressive symptom trajectories, but this led to improper or under-identified solutions, suggesting overparameterization and the need for increased parsimony (Bauer & Curran, 2003). Following guidance from the literature (Diallo, Morin, & Lu, 2017; Morin, McLarnon, & Litalien, 2020; Nylund-Gibson & Masyn, 2016), sociodemographic covariates were included after using fit indices to inform the selection of the optimal variance-covariance structure, functional form, and class solution in unconditional models.

Selection of the optimal class selection was guided by a holistic evaluation of multiple statistics (Masyn, 2013) including Bayesian information criteria (BIC), sample-size adjusted BIC (SSA-BIC; lower is better), and Akaike information criterion (AI); classification quality was evaluated with entropy (values closer to 1 indicates fewer classification errors; van de Schoot, 2017). We prioritized substantive meaningfulness and parsimony during model selection with the aid of visual inspection of trajectories (Muthén, 2003) since fit statistics often yield incongruent results. (Masyn, 2013)

We focused on latent growth mixture models with class-invariant, nondiagonal variance–covariance matrix due to improved fit. Models with five linear trajectory classes had the lowest AIC, BIC, and SSA-BIC; however, the fifth trajectory contained a small group (1% per population) that was comparable to another trajectory (supplement S4-S8). The four-class linear model was selected as the final solution for those with and without military experience based on fit indices, classification quality, and interpretability.

We derived a latent class indicator variable based on individuals’ most likely class membership from the latent class posterior distribution from the best-fitting trajectory model. Next, we added sociodemographic covariates to latent growth mixture models using latent class indicator variables and misclassification error rates (i.e., uncertainty rates). This approach enables the estimation of sociodemographic covariate effects on trajectory class membership in multinomial logistic regression models after accounting for class uncertainty rates (misclassification rates; Vermunt, 2010; Wickrama, Lee, O’Neal, & Lorenz, 2021). Associations were represented by odds ratios (OR) or change in likelihood of membership in one trajectory class versus a comparison trajectory as a function of sociodemographic covariates.

S16

| Sensitivity Analysis: Multinomial Regression Predicting Trajectory Class Membership Relative to Low Depressive Symptoms (LC1) | | | | | | | | | |
| --- | --- | --- | --- | --- | --- | --- | --- | --- | --- |
| SMV (*n*=1,266) | Low/Decreasing (LC2)  vs  Low (LC1) | | | Higher/Declining (LC3)  vs  Low (LC1) | | | Increasing (LC4)  vs  Low (LC1) | | |
|  | OR | *p* | 95% CI | OR | *p* | 95% CI | OR | *p* | 95% CI |
| Male | 1.116 | .653 | [0.691, 1.802] | 0.537 | .149 | [0.231, 1.249] | 0.605 | .151 | [0.305, 1.201] |
| Race (REF = White) | | | | | | | | | |
| Black | 1.047 | .858 | [0.636, 1.722] | 0.619 | .284 | [0.257, 1.489] | 0.520 | .180 | [0.200, 1.352] |
| Latinx | 1.147 | .642 | [0.664, 2.042] | 1.144 | .823 | [0.353, 3.711] | 1.291 | .563 | [0.543, 3.066] |
| Income | 1.099 | .508 | [0.830, 1.455] | 1.264 | .420 | [0.715, 2.233] | 1.176 | .442 | [0.777, 1.780] |
| Mother Education | 1.103 | .774 | [0.564, 2.159] | 1.099 | .841 | [0.437, 2.766] | 0.575 | .251 | [0.223, 1.148] |
| Father Education | 1.122 | .397 | [0.774, 2.107] | 0.510 | .204 | [0.181, 1.440] | 2.126 | .179 | [0.708, 6.382] |
| Family Structure  (REF = 2 Bio Parents) | |  |  |  |  |  |  |  |  |
| Single Parent | 1.345 | .208 | [0.848, 2.135] | 1.624 | .301 | [0.648, 4.070] | 1.319 | .494 | [0.596, 2.916] |
| Stepparents | 0.798 | .468 | [0.434, 1.467] | 1.221 | .768 | [0.324, 4.608] | 1.858 | .183 | [0.747, 4.625] |
| Civilians (*n*=17,644) | OR | *p* | 95% CI | OR | *p* | 95% CI | OR | *p* | 95% CI |
| Male | 0.696 | <.001 | [0.624, 0.775] | 0.423 | <.001 | [0.363, 0.494] | 0.504 | <.001 | [0.386, 0.658] |
| Race (REF = White) |  | | | | | | | | |
| Black | 1.027 | .774 | [0.855, 1.234] | 1.036 | .708 | [0.861, 1.247] | 0.983 | .997 | [0.701, 1.424] |
| Latinx | 1.005 | .963 | [0.829, 1.217] | 1.055 | .557 | [0.882, 1.261] | 0.991 | .955 | [0.712, 1.378] |
| Income | 1.014 | .796 | [0.915, 1.123] | 0.956 | .425 | [0.854, 1.069] | 1.033 | .730 | [0.861, 1.238] |
| Mother Education | 0.870 | .106 | [0.734, 1.030] | 1.052 | .644 | [0.850, 1.301] | 0.795 | .164 | [0.576, 1.098] |
| Father Education | 1.058 | .622 | [0.874, 1.321] | 1.102 | .374 | [0.890, 1.365] | 1.136 | .446 | [0.818, 1.578] |
| Family Structure  (REF = 2 Bio Parents) | |  |  |  |  |  |  |  |  |
| Single Parent | 1.184 | .042 | [1.006, 1.394] | 1.943 | <.001 | [1.650, 2.288] | 1.424 | <.002 | [1.139, 1.781] |
| Stepparents | 0.990 | .928 | [0.803, 1.221] | 1.786 | <.001 | [1.403, 2.273] | 1.381 | .067 | [0.978, 1.951] |

References

Bauer, D. J., & Curran, P. J. (2003). Distributional assumptions of growth mixture models: implications for overextraction of latent trajectory classes. *Psychological Methods*, *8*(3), 338. doi:10.1037/1082-989X.8.3.338

Diallo, T.M.O., Morin, A.J.S., & Lu, H. (2017a). The impact of total and partial inclusion or exclusion of active and inactive time invariant covariates in growth mixture models. *Psychological Methods, 22,* 166-190. doi:10.1037/met0000084

Masyn, K. E. (2013). Latent class analysis and finite mixture modeling. In Todd. D. Little (Ed.), *The Oxford handbook of quantitative methods* (Pp 551-611). Oxford University Press.

Morin, A.J.S., McLarnon, M.J.W., & Litalien, D. (2020). Mixture modeling for organizational behavior research. In Y. Griep & S.D. Hansen (Eds.), *Handbook on the Temporal Dynamics of Organizational Behavior* (pp. 351-379). Cheltenham, UK: Edward Elgar.

Muthén, B. (2003). Statistical and substantive checking in growth mixture modeling: Comment on Bauer and Curran (2003). Los Angeles, CA: Muthén & Muthén.

Nylund-Gibson, K., & Masyn, K.E. (2016). Covariates and mixture modeling: Results of a simulation study exploring the impact of misspecified effects on class enumeration. *Structural Equation Modeling, 23,* 782-797. doi:10.1080/10705511.2016.1221313

Peel, D., & McLachlan, G. J. (2000). Robust mixture modelling using the t distribution. *Statistics and Computing*, *10*, 339-348.

Van De Schoot, R., Sijbrandij, M., Winter, S. D., Depaoli, S., & Vermunt, J. K. (2017). The GRoLTS-checklist: guidelines for reporting on latent trajectory studies. *Structural Equation Modeling: A Multidisciplinary Journal*, *24*(3), 451-467.

Vermunt, J. K. (2010). Latent class modeling with covariates: Two improved three-step approaches. *Political Analysis*, *18*(4), 450-469.

Wood, A.M., Taylor, P. J., & Stephe, J. (2010). Does The CES-D measure a continuum from depression to happiness? Comparing substantive and artifactual models. *Psychiatry Research* 177:120-123. doi:10.1016/j.psychres.2010.02.003

Wickrama, K., Lee, T. K., O’Neal, C. W., & Lorenz, F. (2021). *Higher-order growth curves and mixture modeling with Mplus: A practical guide*. Routledge.
